# Sleep Quality After Intradialytic Oral Nutrition: A New Benefit of This Anabolic Strategy? A Pilot Study

**DOI:** 10.3389/fnut.2022.882367

**Published:** 2022-07-22

**Authors:** Ailema González-Ortiz, Samuel Ramos-Acevedo, Victoria Santiago-Ayala, Gabriela Gaytan, Matilde Valencia-Flores, Ricardo Correa-Rotter, Juan Jesus Carrero, Hong Xu, Ángeles Espinosa-Cuevas

**Affiliations:** ^1^Translational Research Center, Instituto Nacional de Pediatría, Mexico City, Mexico; ^2^Department of Nephrology and Mineral Metabolism, Instituto Nacional de Ciencias Médicas y Nutrición Salvador Zubirán, Mexico City, Mexico; ^3^Neurology Department, Sleep Disorders Clinic, Instituto Nacional de Ciencias Médicas y Nutrición Salvador Zubirán, Mexico City, Mexico; ^4^School of Psychology, Universidad Nacional Autónoma de México, Mexico City, Mexico; ^5^Medical Epidemiology and Biostatistics, Karolinska Institutet, Stockholm, Sweden; ^6^Division of Clinical Geriatrics, Department of Neurobiology, Care Sciences and Society, Karolinska Institutet, Stockholm, Sweden; ^7^Health Care Department, Universidad Autónoma Metropolitana-Xochimilco, Mexico City, Mexico

**Keywords:** intradialytic oral supplementation, sleep quality, hemodialysis, nutrition, anabolic

## Abstract

**Background:**

Since disturbances of appetite and sleep are closely related and both affect metabolic disorders, it would be expected that a renal specific oral nutritional supplement (RS-ONS) that covers the energy the patient does not consume on the HD day, could contribute to improve the nutritional status and body composition, as well as sleep quality. There is still scarce information related to this topic.

**Aim:**

To evaluate the effect of the use of intra-dialytic RS-ONS vs. RS-ONS at home on sleep quality, nutritional status, and body composition in patients on HD.

**Methods:**

Adult patients < 65 years, with ≥3 months on HD were invited to participate in an open randomized pilot study (ISRCTN 33897). Patients were randomized to a dialysis-specific high-protein supplement provided during the HD session (Intradialytic oral nutrition [ION]) or at home (control), during non-HD days (thrice weekly, for both) 12 weeks. The primary outcome was sleep quality defined by the Pittsburgh Sleep Quality Index (PSQI) score. Nutritional assessment included Malnutrition Inflammation Score (MIS), bioelectrical impedance analysis, anthropometry, 3-day food records, and routine blood chemistries.

**Results:**

A total of 23 patients completed the study. Age was median 35 (range 24–48 years), 42% were women. At baseline, the PSQI score was median 4 (range 2–7), and MIS showed a median of 6 (range 5–8); there were no baseline differences between groups. After intervention, both groups improved their MIS scores and similarly when we analyzed the whole cohort (pre- vs. post-intervention *P* < 0.01). Patients in the ION group improved the overall PSQI score to median 3 (2–5), and assessment of sleep duration and sleep disturbances (pre- vs. post-intervention *P* < 0.05), with a trend toward an effect difference compared to patients consuming the supplement at home (P for treatment-effect across arms 0.07 for PSQI score and 0.05 for sleep latency).

**Conclusion:**

Oral supplementation improved nutritional status in the whole cohort, but only ION improved the PSQI score. More studies are needed to explore the nutritional strategies that influence the relationship between sleep and nutritional status in HD patients.

## Introduction

Patients with Chronic Kidney Disease (CKD) undergoing hemodialysis (HD) frequently experience loss of appetite and poor sleep quality, conditions that are closely interrelated. It has been reported that having a good nutritional status and good sleep quality are essential to maintaining quality of life. Previous studies have reported that around 30–80% of patients with advanced CKD had sleep disorders, with a higher prevalence and severity in HD patients (1–3). Furthermore, poor sleep quality has been associated with several health consequences such as metabolic abnormalities, disability, pain, restless leg syndrome, fatigue, sleep apnea, depression, and malnutrition (4).

A decreased nutrient and protein intake due to loss of appetite, a hypercatabolic state, metabolic acidosis, comorbidities, and dialysis itself may all contribute to malnutrition and inflammation also known as protein-energy wasting (PEW) (5), identified as a common problem in patients with CKD, with a current prevalence of 24–54% (6). Treatment of PEW with nutritional strategies such as nutritional support have proven effective and has been associated with adequate tolerance to the supplement and better compliance to the HD treatment (7–9). Interventional and observational studies suggest that nutritional supplementation may have the following benefits: improved quality of life (9), increased body weight and maintenance of lean body mass (10), improved response to erythropoietin (11), improved serum albumin or prealbumin levels (9, 12) better nutritional status (subjective global assessment) (13), increased energy and protein intake (14) and lower mortality (15).

Nutritional guidelines establish the need of nutritional supplementation in order to maintain a minimum energy intake of 25–35 kcal/kg/body weight/day, for those patients with chronically inadequate intake and whose protein and energy requirements cannot be attained by dietary counseling; they suggest a minimum of a 3-month of oral nutritional supplements (16). The use of oral supplementation is considered a therapeutic alternative that can provide 7–10 kcal/kg/day and 0.3–0.4 g/kg/day of protein intake, which helps to meet recommended goals and also may supply a great variety of macro and micro nutrients, as well as covering the skipped meal time, during the day the patient attends a HD session (17).

PEW impacts negatively on CKD patient outcomes, including quality of life, rate of hospitalizations, presence and severity of infections, cardiovascular events, survival (5, 7, 8), and sleep quality (9). It has been demonstrated that PEW can be treated *via* oral supplementation (16). It has been known that HD therapy has been associated with net protein loss from the whole body, however, this catabolic process can be reversed by oral nutrition during HD session (18). In addition, the anabolic effects of intradialytic oral nutrition (ION) seem not to be limited to the administration period like parenteral nutrition (18). However, despite the evidence, there is no clear indication of the best time to use oral supplementation (16). The use of a nutritional supplement would allow, in the first instance, to cover both energy and protein intake in this population and, in turn, reduce protein catabolism caused by dialysis treatment, so that its use during the HD session may be even more effective (18). On the other hand, sleep disturbances are increasingly being studied and associated with the intake of both macro and micro nutrients, mainly in the general population, where hours of sleep have been associated with different nutritional outcomes (19). Hemodialysis patients often have frequent sleep disturbances, as well as poor appetite, malnutrition, and body composition (20, 21).

According to this, the purpose of this study was to evaluate the effect of the use of renal specific ION supplementation (RS-ONS) vs. RS-ONS at home on sleep quality, nutritional status, and body composition in patients on HD.

## Materials and Methods

### Study Population

We performed a single center, open randomized pilot study for prevalent patients undergoing HD in our unit. The study was approved by the institutional ethics committee (registration number 2229), in addition to having the registration of International Standard of Randomized Controlled Trials Number (ISRCTN 33897). Eligible participants were adults (>18 years), both sexes, under maintenance HD (at least 3 months on therapy), thrice weekly, and Kt/V >1.2 or URR >65%. Patients with (1) amputation of any extremity, (2) planned renal transplant within the next 3 months, (3) acute kidney injury, hospitalization 1 month prior to the initiation of the study, and (4) those who had ultrafiltration volumes of more than 3 liters per session or with sleep disorders (diagnosed by sleep clinic experts) were excluded. All patients were informed about the nature of the study and signed an informed consent. Simple randomization to one of two groups was performed by an external collaborator without contact with the research team in charge of enrollment and intervention using a website (randomizer.org).

### Dietary Assessment

All patients were given personalized nutritional counseling, according to current guidelines (22, 23). Dietary intake was evaluated by 3-day food records, asking the patient to record food intake on a HD day, a non-HD mid-weekday, and a weekend day. For this purpose, patients received training on how to record their food consumption by a trained dietitian. Records were reviewed with the patient and corrected with the help of standardized tridimensional and flat food replicas. Food records were introduced into the software Nutrikcal VO® v.1, which determines the energy and macronutrients provided by each food group, according to Mexican guidelines and food composition of typical Mexican foods (24).

### Intradialytic Oral Nutrition

**Figure 1:**
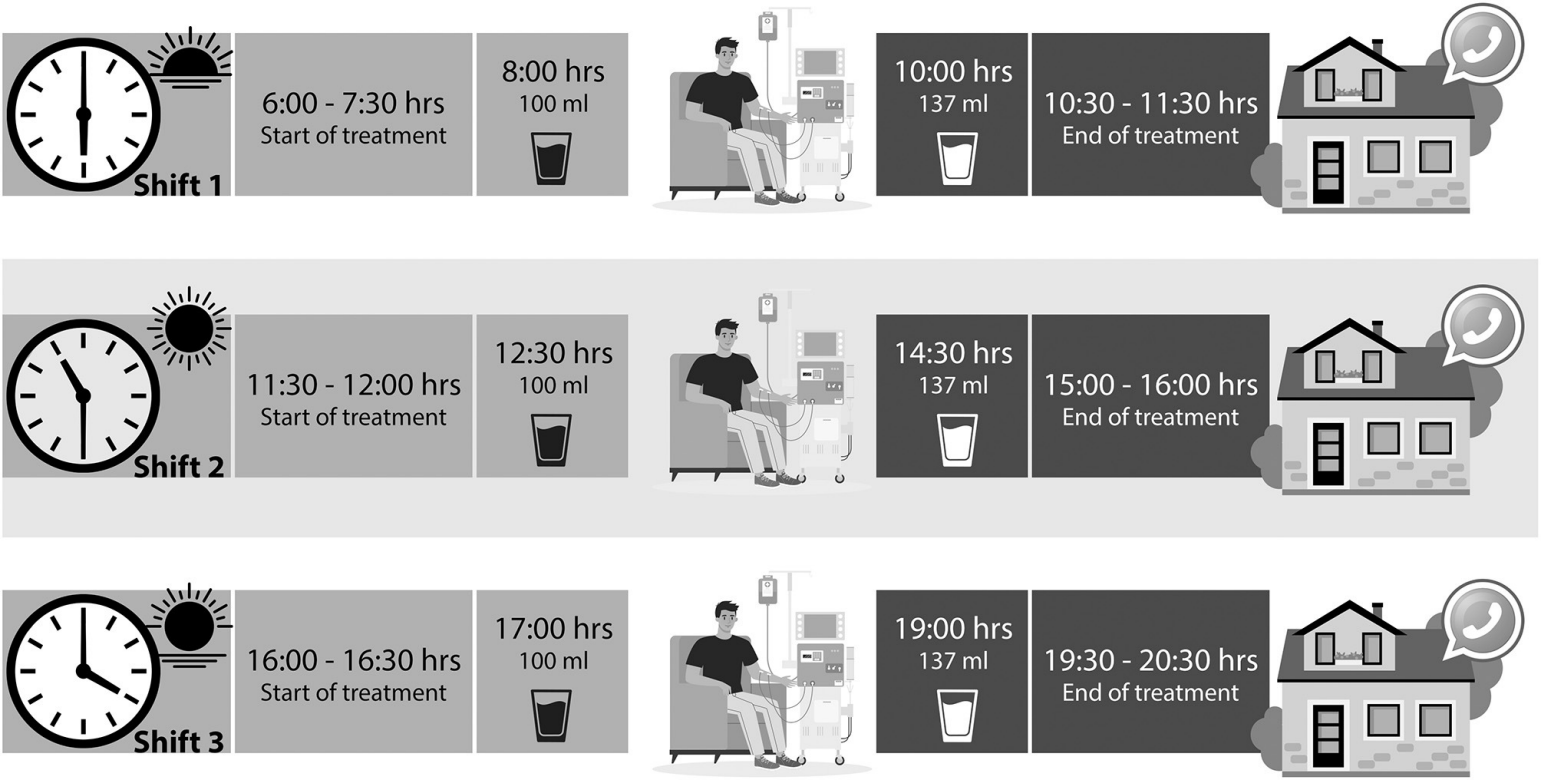
Study protocol. Supplement intake according to the treatment schedule, for patients who took it at home a message was sent to remind them to take it at the time they come to HD.

The patients in this group received two portions of the RS-ONS in plastic recipients with a lid and straw; the first portion was 100 ml and was received at minute 60, and the second portion was 137 ml and was received 45 min before ending their sessions. The RS-ONS was Nepro? (Abbott Laboratories) with the following nutritional content in 237 ml: 434 kcal, 37.9 g of carbohydrates, 22.8 g of lipids, 19.2 g of protein, and 3 g of fiber in 237 ml (1.83 kcal/ml). Cans were washed with 2.0% wide spectrum chlorhexidine, following appropriate guidelines. The intervention was provided over 36 consecutive HD sessions (12 weeks, 3 weekly). If a patient missed a session, the respective supplement was labeled with a code and saved until the end of the study (Figure 1).

### Supplementation at Home

The participants in this group received the same RS-ONS on a non-dialysis day at home (thrice weekly for 12 weeks). They were encouraged to consume their can in two portions, following the suggested schedule (as if they had attended the session, at the same time as the intradialytic group) and wash the canned product before consuming it. The patients were asked to return the empty can on the following HD day so they could receive the next can (Figure 1).

### Study Outcomes

#### Sleep Quality

The assessment of sleep pattern and quality was performed by a sleep specialist before a HD session with the Pittsburgh Sleep Quality Scale (PSQI), which is a self-report tool of 19 items regarding sleep quality and degree of difficulty sleeping during the last month. It is made up of 7 components: sleep quality, sleep latency, sleep duration, sleep efficiency, sleep disturbances, use of sleeping medications, and daytime dysfunction. The 7 components are added to produce an overall score with a range from 0 to 12; higher scores indicate poorer sleep quality. An overall score >5 has a diagnostic sensitivity of between 89.6 and 98.7%, and specificity of 84.4–86.5%, differentiating between bad and good sleepers (25, 26).

### Nutritional Status

The Malnutrition Inflammation Score (MIS) calculation method includes (A) medical history: Changes in dry weight after dialysis (3–6 months), dietary intake, gastrointestinal symptoms, functional capacity, and comorbidity according to time on HD. (B) Physical exam, loss of fat stores or subcutaneous fat below eyes, triceps, biceps, chest, and signs of loss of lean mass in temple, clavicle, scapula, ribs, quadriceps, knee, interosseous, (C) body mass index (BMI), and (D) biochemical parameters such as serum albumin and total iron binding capacity (TIBC) or transferrin, which are estimated from the results of the various evaluations. Each of them is classified into four degrees of severity. Starting from 0 to 3 on the scale, the sum of all 10 MIS components can range from 0 (normal) to 30 (severely malnourished)—a higher score reflects a more severe degree of malnutrition and inflammation (27).

### Body Composition

The participants were evaluated at the end of the HD session at the following time points: at baseline, and at the end of the follow-up—we included body weight and height to determine BMI. For the measurement of bioelectrical impedance analysis (BIE), two “emitting electrodes and two sensor electrodes” were carefully placed (standard, tetrapolar placement on the hand and foot). Measurements were conducted by the same operator using an impedance device that emitted 800 μA and 50 kHz alternating sinusoidal current (Multifrequency Bodystat Quadscan 4000 BODYSTAT Ltd.), strictly following the methods reported elsewhere (28).

### Study Covariates

Other study covariates were collected *via* standardized methods and included demographics such as self-reported civil status, employment, comorbidities, medications, and laboratory measurements. History of comorbidities and ongoing medications were obtained from consultation of the patient's clinical files. Kt/V as a measurement of dialysis adequacy (K = dialyzer urea clearance, t = time on dialysis, and V = total body water) was extracted from the medical records which was calculated by the machine (Fresenius Medical Care 4008 s) at the end of the sessions. All measurements were carried at baseline and 12 weeks after the completion of the nutritional intervention.

### Sample Size

Since there are no previous studies that have evaluated the effect of ION supplementation on sleep quality, we analyzed the entire population of our HD unit, and subsequently performed a statistical power analysis. Power of the sample size was calculated using GPower^19^® (version 3.1.9.4; Heinrich-Heine-University, Düsseldorf, Germany). A statistical power calculation was made with the included sample size in which the effect size was 0.50, giving a total power of 0.608 with two tails considering the whole cohort.

### Statistical Analysis

Descriptive statistics were performed, and continuous variables were presented as arithmetic means and standard deviation for those that had a normal distribution, and those that did not were presented as medians or the inter-quartile range. We carried out analysis following protocol, and to determine whether there were statistical differences between the groups, the chi-square test was performed for the qualitative variables. For the quantitative, the Mann Whitney U tests or the Student's *t*-test was employed, depending on their distribution. Wilcoxon or paired *T-*tests were used to determine the differences between the basal and final groups, according to the distribution of the data.

A subanalysis was performed by group and in all the patients as one group.

The analyses were performed using STATA (version 15.1; Stata Corp, College Station, TX) and *p* ≤ 0.05 was established for statistical significance.

## Results

**Figure 2:**
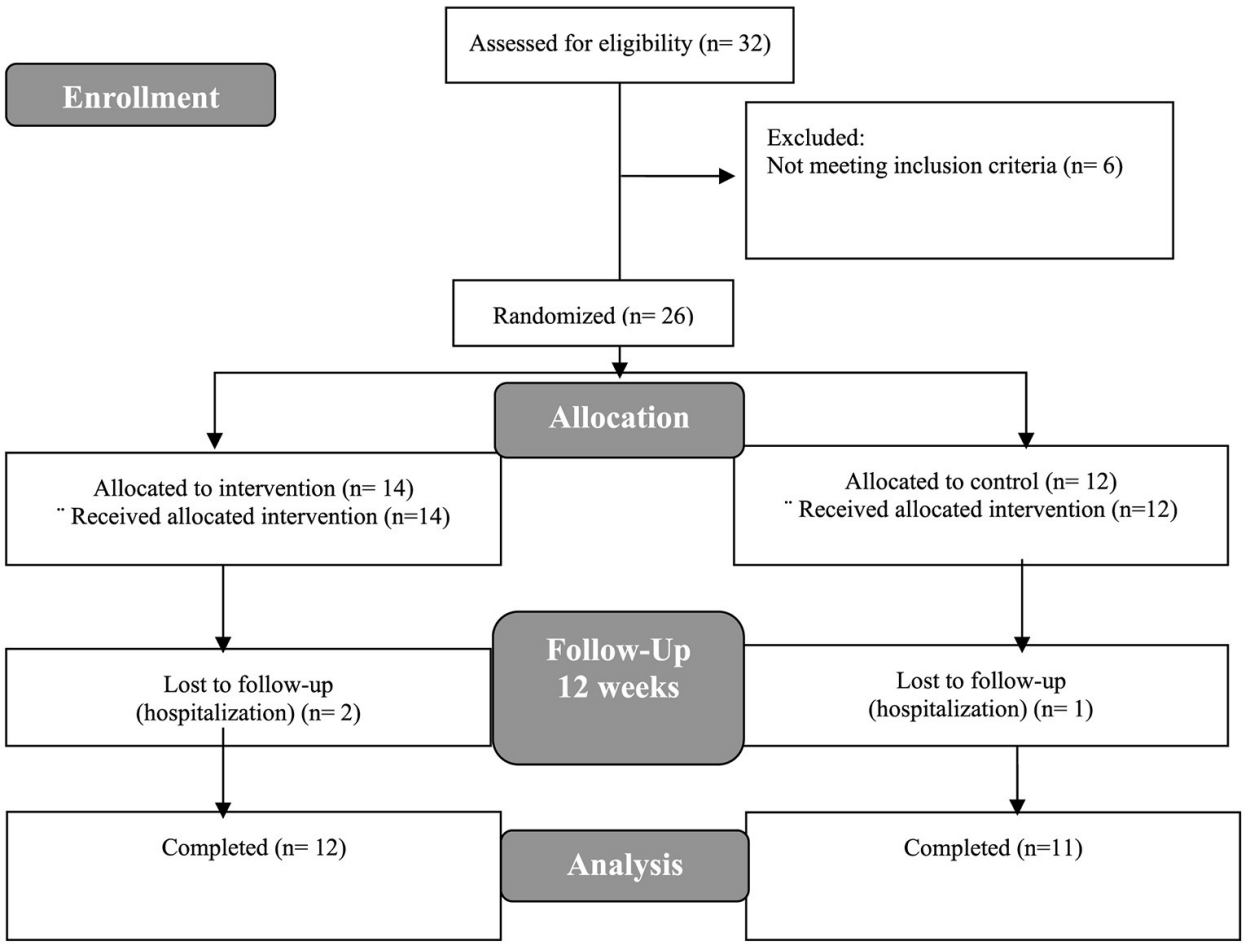
Flowchart of study design.

A total of 26 participants were randomized, of which 23 finished the study; in general, 94% adherence to treatment was observed. The description of patient selection and monitoring are explained in detail in Figure 2. This was a relatively young population with almost half of them with diabetes mellitus. Regarding biochemical measurements, a median of 3.6 (range 3.2–3.7) g/dl of serum albumin was observed, while iron profiles were in normal ranges. There were no differences at baseline in dietary energy and protein intake between groups (Table 1), nor in the whole cohort comparing baseline and final measurements (data not shown).

**Table 1:** General characteristics at baseline.

**Characteristic**	**All (*n* = 26)**	**At home (*n* = 12)**	**ION (14)**	***P*** **-Value**
PSQI total score	4 (2–7)	3 (2–6)	6.5 (3–11)	0.06
Age (years)	35 (24–48)	33.5 (24–44)	36 (30–49)	0.59
Dialysis vintage (months)	16 (8–36)	12 (7–33)	21.5 (8–40)	0.66
Gender, women *n* (%)	11 (42)	6 (50)	5 (36)	0.46
Civil status single, *n* (%)	13 (50)	7 (58)	6 (43)	0.36
Diabetes, *n* (%)	12 (46)	5 (42)	7 (50)	0.67
Hypertension, *n* (%)	22 (85)	11 (92)	11(79)	0.36
**Biochemical measurements**				
Albumin (g/L)	3.6 (3.3–3.7)	3.95 (3.5–4.3)	3.45 (3.2–3.7)	0.05
Serum Iron (μg/dL)	56 (44–74)	54 (42.5–57)	62 (46–82)	0.22
Unsaturated iron binding capacity (μg/dL)	214 (186–256)	230.5 (204–257)	187 (181–248)	0.11
Total iron binding capacity (μg/dL)	256 (237–315)	273.5 (252.5–302.5)	250.5 (227–330)	0.32
Saturation index %	23 (16–34)	20 (14.5–28.5)	25 (19–34)	0.27
Ferritin mg/dL	146 (50.5–330.4)	145.6 (35.4–262.2)	145.6 (89.4–348.5)	0.66
Serum creatinine (mg/dL)	11.4 (10–12.9)	11.4 (10.3–13.1)	11.2 (10–12.9)	0.96
Sodium (mmol/L)	139 ± 2.5	139 ± 2.6	139 ± 2.4	0.79
Serum potassium (mmol/L)	5.2 (5.1–5.6)	5.2 (5.1–5.4)	5.3 (5.1–5.7)	0.69
Serum phosphorus(μg/L)	4.4 (3.4–5.6)	4.1 (3.5–5.3)	4.6 (3.4–7.3)	0.59
Total KT/V	1.9 ± 0.5	1.8 ± 0.3	1.99 ± 0.7	0.44
**Dietary intake and food groups (by 1,000 kcal) per day**				
Energy, kcal	1,397 (1,087–1,850)	1,302 (1,087–1,628)	1,427 (1,240–1,850)	0.30
Dietary energy intake, kcal/kg/day	22.8 (16.5–30.5)	20.6 (16.5–28.2)	23.7 (20.5–30.5)	0.26
Protein g/kg/day	0.96 (0.64–1.14)	0.83 (0.64–1.12)	0.97 (0.91–1.14)	0.28
Fiber g/day	8.9 (6.3–10.7)	8.4 (6.3–10.7)	8.9 (7.6–9.2)	0.92
Fruits (servings)	0.8 (0.6–1.8)	0.7 (0.6–1.8)	0.8 (0.6–0.96)	0.94
Vegetables (servings)	1.4 (0.9–1.9)	1.4 (1.1–1.7)	1.5 (0.9–1.9)	0.84
Legumes (servings)	0.07 (0–0.5)	0.03 (0–0.16)	0.08 (0.03–0.22)	0.32
Cereals (servings)	5.6 (4.5–6.1)	5.6 (5.0–6.1)	5.6 (4.5–5.9)	0.88
Fat (servings)	3.5 (2.3–5.3)	3.5 (2.3–4.8)	3.6 (2.9–5.3)	0.84
Dairy (servings)	0.1 (0–0.5)	0.14 (0–0.5)	0.13 (0–0.4)	0.98
Meat and eggs (servings)	3.6 (2.6–4.7)	3.5 (2.8–4.2)	3.7 (2.6–4.7)	0.96

*Data are expressed as mean ± SD, median (25th, 75th centile), or number (%), as appropriate*.

### Sleep Quality

The basal median of the total sample on PSQI was 4 (range 2–7) points. Sleepiness was present in 8% of the sample at baseline and 14 patients (46%) were poor sleepers (PSQI > 5).

Subsequently, after 12 weeks of follow-up, the Pittsburgh components were analyzed between groups, and a significant decrease difference was found in the sleep latency, as well as in the presence of sleep disturbances (*p* < 0.05 for both groups). The ION group showed improvement in the sleep duration and disturbances as well as a decreased prescription of sleep medications. The total score improved in the ION group (pre- vs. post-intervention, *p* < 0.05), whereas no differences were found in the home group (pre- vs. post-intervention) (Table 2). When we explored the difference in sleep changes between groups we found a statistical difference only in sleep latency in the ION group, with a trend toward improvement in the sleep disturbances and overall PSQI scores (Table 3).

**Table 2:** Effects of ION on sleep quality index components, nutritional status, and body composition.

	**At home**		**ION**	
**Characteristics**	**Baseline**	**Final**	**Baseline**	**Final**
	***n*** **= 11**	***n*** **= 11**	***n*** **= 12**	***n*** **= 12**
**Pittsburg sleep quality index components**				
Subjective sleep quality	1 (0–1)	0 (0–1)	1 (1–2)	1 (0–1)
Sleep latency	0.5 (0–1)	0 (0–1)	1.5 (0–3)	1 (0.5–1)
Sleep duration	0 (0–2)	0 (0–1)	1 (0–2)	0 (0–1)^*^
Habitual sleep efficiency	0 (0–1)	1 (0–1)	0.5 (0–2)	0 (0–1)
Sleep disturbances	1 (0–1)	1 (0–1)	1 (1–2)	1(0.5–1)^*^
Daytime dysfunction	0 (0–0)	0 (0–0)	0 (0–0)	0 (0–0)
Use of medication for sleep	0 (0–1)	1 (0–0)	0.5 (0–1)	0 (0–0)^*^
PSQI total score	3 (2–6)	3 (1−6)	6.5 (3–11)	3 (2–5)^*^
Poor sleeper, *n* (%)	4 (33)	2 (18)	8 (57)	1 (8)
Sleep hours	7.5 (5–8)	8 (6.5–8)	7 (5–8)	8 (7–9)^*^
**Nutritional status and body composition**				
Body mass index (kg/m^2^)	24 (20–29)	23 (20–30)^*^	22 (20–27)	23 (21–28)^*^
nPNA (g/kg/day)	0.89 (0.83–1.13)	1.06 (0.95- 1.12)^*^	0.9 (0.79–1.01)	1.0 (0.9–1.13)
Resistance/height^*^	354.5 (310–422)	359 (302–454)	355.4 (329-468)	324 (291 - 429)^*^
Reactance/height^*^	42 (32–49)	38 (34–44)	41 (35–50)	40 (33.6–45.5)
Phase angle (degres)^**^	5.9 ± 1.2	6.1 ± 0.9	6.15 ± 1.13	6.4 ± 1.1
**Malnutrition inflammation score components**				
Medical history	3 (0–6)	1 (0–4)	2.5 (1–6)	2 (1–3)^*^
Physical exam	2 (0–4)	0 (0–2)^*^	1.5 (0–3)	0 (0–1)^*^
Body mass index	0 (0–2)	0 (0–2)	0 (0–2)	0 (0–2)
Laboratory parameters	1 (0–2)	1 (0–2)	2 (0–4)^*^	1 (0–4)
Total MIS score	6 (2–11)	2.5 (1–8)^*^	6.5 (2–10)	4 (2–6)^*^

*Wilcoxon rank-sum (Mann-Whitney test). Data are expressed as mean ± SD, median (25th, 75th centile), or number (%), as appropriate. MIS, Malnutrition Inflammation Score*.

* *Pre- vs. post-intervention p < 0.05*.

** *At 50kHz*.

*Medical history represents the sum of score: change in dry weight, dietary intake, gastrointestinal symptoms, functional capacity and co-morbidity*.

*Physical exam represents the sum of score: decreased fat stores or loss of subcutaneous fat and signs of muscle wasting*.

*Laboratory parameters represents the sum of score: albumin and total iron binding capacity*.

**Table 3:** Changes (Δ) in sleep quality characteristics, nutritional status, and body composition in both study groups.

**Sleep characteristics**	**At home**	**ION**	***P*** **-Value**
	**(Δ) (*n* = 11)**	**(Δ) (*n* = 12)**	
Sleep hours	0.5 (-1.5–3)	0.75 (-1–3)	0.42
**PSQI components**			
Subjective sleep quality	0 (-2–2)	0 (-2–1)	0.68
Sleep latency	0 (-1–2)	-0.5 (-2–1)	0.05
Sleep duration	0 (-2–1)	-0.5 (-2–1)	0.53
Habitual sleep efficiency	0 (-1–2)	0.5 (-3–1)	0.13
Sleep disturbances	0 (-1–1)	0 (-2–0)	0.08
Daytime dysfunction	0 (0–0)	0 (-1–0)	0.34
Use of medication for sleep	0 (-1–1)	0 (-1–0)	0.28
PSQI total score	1 (-5–6)	-2.5 (-10–2)	0.07
**Nutritional status and body composition**			
Body mass index kg/m^2^	0.75 (-0.9–1.7)	0.89 (-0.04–2.0)	0.19
MIS score	-2 (-9–0)	-3 (– 8–0)	0.45
nPNA (g/kg/day)	0.09 (-0.09–0.32)	0.15 (-0.42–0.53)	0.93
Resistance/height^*^	-7.8 (-60.6–145.2)	-34.5 (-66.6–23.9)	0.04
Reactance/height^*^	-1.9 (-30.6–14.9)	-3.1 (-13.3–8.7)	0.79
Phase angle (degrees)^*^	0.2 (-1.91–2.4)	0.3 (-1.9–1.84)	0.92
Albumin mg/dL	0 (-0.4–0.36)	0 (0–1.6)	0.09

*Wilcoxon rank-sum. Data are expressed as median (25th, 75th centile), or number (%), as appropriate*.

* *At 50 kHz*.

**Figure 3:**
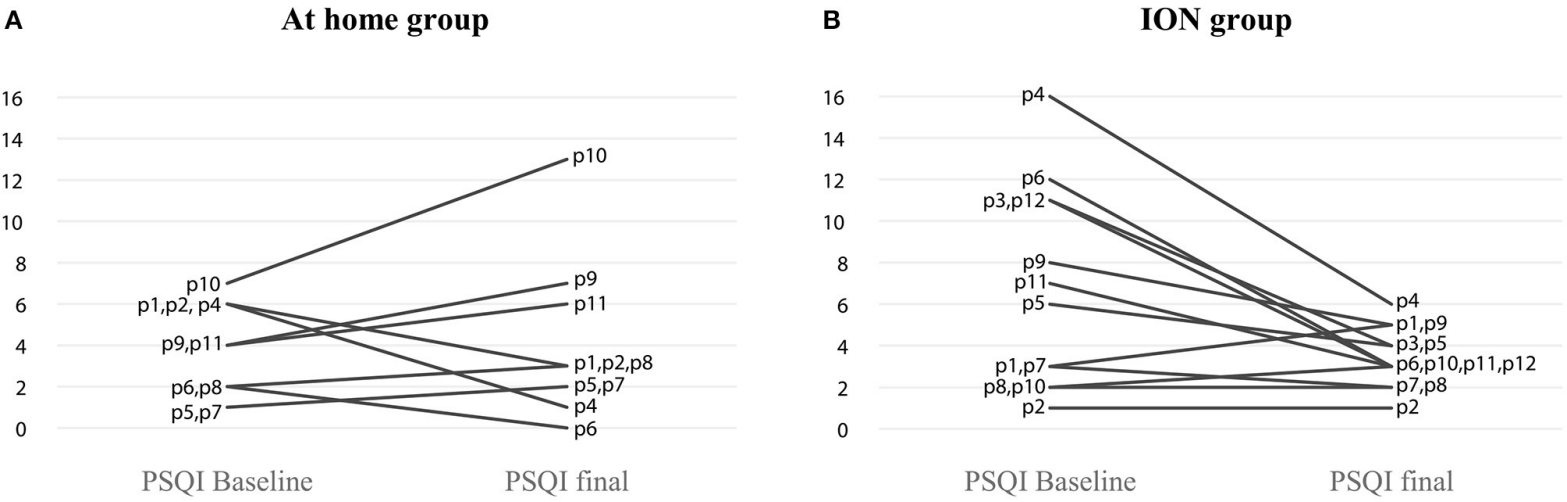
Change in Pittsburgh score after 12 weeks of oral supplementation. **(A)** At home group and **(B)** ION group.

When we evaluated the Pittsburgh score individually for each patient, we observed a trend to improve mainly in those of the ION group (Figure 3B), while the group that received supplementation at home, the score seemed to worsen by the end of the intervention (Figure 3A). Considering a decrease of two points in the PSQI an improvement, seven participants (58%) improved in the ION group; the number needed to treat indicated that we must treat eight [CI 95% (6.9–8.6)] patients with ION to decrease two points in the PSQI.

### Nutritional Status and Body Composition

After randomization, no differences were found between groups. At the end of the study, we found an improvement in MIS score in both groups, pre- vs. post-intervention (*p* < 0.05), but no statistically significant differences were observed between the groups. In the ION group, BMI showed improvement; however, in the home group, the BMI decreased and nPNA improved after intervention, while no other differences were observed (Table 2). No differences were observed between groups in other nutrition status markers. Finally, considering it as an improvement of nutritional status, a decrease of two points was observed in the MIS score; almost all participants improved-−10 participants (83%) in the ION group and 8 participants (73%) in the home group. The number needed to treat indicated that we must treat 10 patients with ION to decrease two points in MIS 4.

When we analyzed the whole cohort as one group, we found a decrease in unsaturated iron-binding capacity, no other differences in biochemical measurements. According to PSQI components, we found an improvement in sleep duration and in the total score, when we classified poor vs good sleepers at the end of the study only 3 participants were poor sleepers, and the time to sleep increased, and according to MIS, only BMI not shown differences at the end of the study (Table 4).

**Table 4:** Effects of ION on sleep quality index components, nutritional status, and body composition.

**Characteristics**	**Baseline**	**Final**	***P*** **-value**
	**(*n* = 23)**	**(*n* = 23)**	
Albumin (g/L)	3.5 (3.3–4.3)	3.7 (3.5–4.5)	0.44
Serum Iron (μg/dL)	56 (44–74)	59.5 (46–74)	0.17
Unsaturated iron binding capacity (μg/dL)	214 (186–256)	194 (137–239)	0.02
Total iron binding capacity (μg/dL)	256 (237–315)	254 (224–292)	0.41
Saturation index %	23 (15–34)	21 (16–35)	0.85
Ferritin mg/dL	146 (50.5–334.4)	243.5 (98–395)	0.10
Serum creatinine (mg/dL)	11.6 (10.4–13.52)	10.8 (9.7–12.1)	0.09
Sodium (mmol/L)	139 ± 2.5	138 ± 3.13	0.25
Serum potassium (mmol/L)	5.2 (5.1–5.6)	5.1 (4.7–6)	0.76
Serum phosphorus(μg/L)	4.4 (3.7–5.9)	5.1 (3.9–5.8)	0.91
Total KT/V	1.9 ± 0.5	1.8 (1.5–1.93)	0.24
**Pittsburg sleep quality index components**			
Subjective sleep quality	1 (0–01)	1 (0–1)	0.16
Sleep latency	1 (0–2)	1 (0–1)	0.56
Sleep duration	1 (0–2)	0 (0–1)	0.02
Habitual sleep efficiency	0 (0–2)	0 (0–1)	0.25
Sleep disturbances	1 (1)	1 (0–1)	0.08
Daytime dysfunction	0 (0)	0 (0)	0.32
Use of medication for sleep	0 (0–1)	0 (0)	0.06
PSQI total score	4 (2–7)	3 (2−5)	0.05
Poor sleeper, *n* (%)	11 (48)	3 (13)	0.02
Sleep hours	7 (5–8)	8 (6.5–8.5)	<0.01
**Nutritional status and body composition**			
Body mass index (kg/m^2^)	22 (20–30)	23 (20-31)	<0.01
nPNA (g/kg/day)	0.93 (0.8–1.04)	1.03 (0.88–1.12)	<0.05
Resistance/height^*^	358 (320–445)	335 (284–434)	0.03
Reactance/height^*^	41(32–49)	38 (34–44)	0.17
Phase angle (degres)^**^	6 ± 1.19	6.2 ± 0.89	0.63
**Malnutrition inflammation score components**			
Medical history	3 (2–4)	2 (1–2)	<0.01
Physical exam	2 (0–3)	0 (0–1)	<0.01
Body mass index	0 (0)	0 (0)	0.32
Laboratory parameters	1 (1–2)	1 (0-2)	<0.01
Total MIS score	6 (5–8)	3 (2–4)	<0.01

*Wilcoxon rank-sum (Mann-Whitney test). Data are expressed as mean ± SD, median (25th, 75th centile), or number (%), as appropriate. MIS, Malnutrition Inflammation Score*.

* *Pre- vs post-intervention p value < 0.05*.

** *At 50 kHz*.

*Medical history represents the sum of score: Change in dry weight, dietary intake, gastrointestinal symptoms, functional capacity and co-morbidity*.

*Physical exam represents the sum of score: decreased fat stores or loss of subcutaneous fat and signs of muscle wasting*.

*Laboratory parameters represents the sum of score: albumin and total iron binding capacity*.

## Discussion

Information regarding the use of oral supplements and the effect on sleep in HD patients is scarce, however, there is a large body of evidence that associates dietary intake with sleep quality in the general population, where the main outcomes are focused on nutritional status. This study suggests that intradialytic oral nutrition may improve sleep quality and both interventions (at home and nutrition supplementation during HD sessions) were effective in improving nutritional status.

When we analyzed sleep results, an effect was found only in the group that received ION. There is evidence linking the nutritional status assessed by the MIS score and sleep quality, where those patients who had the worst sleep quality were also those who had the worst nutritional status (29). Therefore, it was expected that those who would improve their nutritional status after reported using a nutritional supplement would also improve their sleep quality; however, it was observed that through this intervention, only the ION group showed improvement.

There are other studies that associate nutritional status with sleep quality in this population. Burrowes et al. (30) found a decreased appetite as sleep quality worsened. In this study, after ION improved patients' sleep quality, however, no differences were found in diet characteristics. At the same time, it has been reported that higher serum creatinine values are associated with better sleep quality, and that this has also been considered as a factor that indicates better muscle mass as nutritional status is variable.

Recent research has shown an association between dietary intake and sleep health that can influence risk factors for chronic diseases. In a recent review, it was described that meal time and sleep hygiene are two of the most important aspects to investigate in the link between diet and sleep (19). And in hemodialysis patients has been studied also how the patients use to skip meal on dialysis day (20).

The presence of sleep disturbances is highly prevalent in this population and dialysis modality or age can be important factors (31, 32). Both, the presence of sleepiness and poor sleep quality have been associated with worse quality of life of these type of patients (33) as this is a persistent problem; however, in this study, the presence of sleepiness was observed only in two individuals, so it is not possible to know if the improvement in the nutritional status had any effect in this parameter (data not shown).

When we evaluated sleep quality characteristics, the group receiving ION presented improvement, mainly in terms of sleep duration and sleep disturbances as well as in a reduction of use of sleep medications (*p* < 0.05 intra-group effect). Similarly, there was an effect on the total PSQI score in the ION group. No effects were found between supplementation at home and sleep quality nor when exploring the difference between the groups. But, when we analyzed the whole cohort the results shown an improvement in sleep duration, sleep hours, fewer poor sleepers, and lower PSQI total score.

It is important to mention that this finding not only supports the use of an oral nutritional supplement in HD patients for nutritional improvement, but could also imply an improvement in sleep quality, if administered during the HD session. When we analyzed the results individually, we observed that a large proportion of participants in the ION group presented a decrease in the PSQI score, while the patients in the home group seemed to have a worse score. Although ION seems to have an effect on sleep quality in this study, it is clearly necessary to consider conducting clinical trials with a larger sample size. Nevertheless, this is one of the few studies that explore the relationship between ION and quality of sleep.

There are multiple proven benefits regarding the use of ION in HD patients; it has been shown to have persistent anabolic benefits for muscle protein metabolism in the post-HD phase, while the anabolic benefits of parenteral nutrition during HD sessions dissipated during the same period. These data support both the anabolic and anti-catabolic functions of ION (18). However, in this study, we observed that the use of a nutritional supplement is effective in both groups—patients who consume it during the HD session and those who had the supplementation at home. This was evidenced through the results obtained by the MIS score that improved significantly in both groups (*p* < 0.05, without statistical differences between the groups), and this is consistent when we analyzed the whole cohort, were we found improvements in all the components an exception of BMI, which was already normal at the beginning of the study. One explanation for these positive results could be adherence to treatment, which was higher than 90% in all the population.

There is a large body of evidence that states that oral supplementation decreases hospital admission rates, serum IL-6 levels (34), improves hypoalbuminemia (35), physical functionality (36), PEW (37), better body composition markers (38), quality of life (39), and reduces mortality (40). However, the evidence of ION compared with nutrition at home is scarce, although this strategy has already been shown to be safe and effective, as recently demonstrated by Ramos-Acevedo et al. (41).

On the other hand, BMI has been reported to be a nutritional status factor that can be maintained regardless of the supplement used during dialysis, as recently reported in a 6-month follow-up clinical trial, comparing consumption of a normal meal vs. a hyper-protein meal intra-dialysis; both groups maintained BMI but not for albumin, where the results were better for the group that received the hyper-protein meal (38).

Martin-Alemañy et al. demonstrated the combined effect of oral supplementation plus aerobic or resistance exercise on nutritional status, and physical functionality (42). Ocepek et al. found that in malnourished patients who had previously received oral supplementation but were not presently receiving them, serum albumin and hand-grip strength tended to worsen, and even for those who were well-nourished, the nutritional markers decreased (43), indicating, as expected, that the effect is not permanent. So, it should be done constantly, including the entire population as a part of a daily practice in HD patients.

A major limitation of our study is our small sample size and its low mean age, which may not reflect the usual on dialysis in most countries; however, this is the first trial that associates sleep quality with the use of oral supplementation. The use of oral supplementation improves nutritional status but it is necessary to perform more RCTs with larger sample sizes to explore the mechanisms that influence the relationship between ION and sleep quality, and is necessary to implement strategies to help compliance for a long time.

A clinical application of this paper is that oral supplementation has been studied in different outcomes, however, studies about sleep outcomes are missing.

In Conclusion, the results of this pilot study support the implementation of oral supplementation, as a strategy to improve nutritional outcomes and that could have an effect on sleep quality.

## Data Availability Statement

The raw data supporting the conclusions of this article will be made available by the authors, without undue reservation.

## Ethics Statement

The studies involving human participants were reviewed and approved by Comité de ética, Instituto Nacional de Ciencias Médicas y Nutrición Salvador Zubirán #2229. The patients/participants provided their written informed consent to participate in this study.

## Author Contributions

AG-O and SR-A participated in study conception, design the research generation, analysis of the data, and writing the paper. ÁE-C participated in study conception and design, revision, analysis of the data, writing the paper, and approval of the final version of the manuscript. VS-A, GG, RC-R, MV-F, HX, and JJC participated in interpretation of the data and/or critical revision of the manuscript to its final form. All authors read and approved the final manuscript.

## Funding

AG-O and SR-A were supported by The National Council of Science and Technology (CONACYT), CVU 373297 and 779601, respectively.

## Conflict of Interest

JJC reports grant funding from AstraZeneca, ViforPharma and Astellas, consulting for Baxter and AstraZeneca, speaker fees for Abbott, Nutricia, AstraZeneca, and ViforPharma all outside the submitted work. ÁE-C acknowledges speaker honoraria from Abbott Laboratories and AbbVie. AG-O acknowledges being speaker for Abbott Laboratories. RC-R reports grant funding from AstraZeneca, Novonordisk, and Glaxo. Consulting fees for Boheringer Ingelheim, AstraZeneca, Chinook, and all unrelated to the submitted work. The remaining authors declare that the research was conducted in the absence of any commercial or financial relationships that could be construed as a potential conflict of interest.

## Publisher's Note

All claims expressed in this article are solely those of the authors and do not necessarily represent those of their affiliated organizations, or those of the publisher, the editors and the reviewers. Any product that may be evaluated in this article, or claim that may be made by its manufacturer, is not guaranteed or endorsed by the publisher.
